# A natural language processing approach to support biomedical data harmonization: Leveraging large language models

**DOI:** 10.1371/journal.pone.0328262

**Published:** 2025-07-24

**Authors:** Zexu Li, Suraj P. Prabhu, Zachary T. Popp, Shubhi S. Jain, Vijetha Balakundi, Ting Fang Alvin Ang, Rhoda Au, Jinying Chen

**Affiliations:** 1 Department of Anatomy and Neurobiology, Boston University Chobanian & Avedisian School of Medicine, Boston, Massachusetts, United States of America; 2 Department of Bioinformatics, Boston University Faculty of Computing & Data Sciences, Boston, Massachusetts, United States of America; 3 Slone Epidemiology Center, Boston University Chobanian & Avedisian School of Medicine, Boston, Massachusetts, United States of America; 4 Department of Medicine/Section of Preventive Medicine and Epidemiology, Boston University Chobanian & Avedisian School of Medicine, Boston, Massachusetts, United States of America; 5 Framingham Heart Study, Boston University Chobanian & Avedisian School of Medicine, Boston, Massachusetts, United States of America; 6 Department of Neurology, Boston University Chobanian & Avedisian School of Medicine, Boston, Massachusetts, United States of America; 7 Department of Epidemiology, Boston University School of Public Health, Boston, Massachusetts, United States of America; 8 Department of Medicine/Section of Genetics, Boston University Chobanian & Avedisian School of Medicine, Boston, Massachusetts, United States of America; 9 Data Science Core, Boston University Chobanian & Avedisian School of Medicine, Boston, Massachusetts, United States of America; Wilfrid Laurier University - Waterloo Campus: Wilfrid Laurier University, CANADA

## Abstract

**Background:**

Biomedical research requires large, diverse samples to produce unbiased results. Retrospective data harmonization is often used to integrate existing datasets to create these samples, but the process is labor-intensive. Automated methods for matching variables across datasets can accelerate this process, particularly when harmonizing datasets with numerous variables and varied naming conventions. Research in this area has been limited, primarily focusing on lexical matching and ontology-based semantic matching. We aimed to develop new methods, leveraging large language models (LLMs) and ensemble learning, to automate variable matching.

**Methods:**

This study utilized data from two GERAS cohort studies (European [EU] and Japan [JP]) obtained through the Alzheimer’s Disease (AD) Data Initiative’s AD workbench. We first manually created a dataset by matching 347 EU variables with 1322 candidate JP variables and treated matched variable pairs as positive instances and unmatched pairs as negative instances. We then developed four natural language processing (NLP) methods using state-of-the-art LLMs (E5, MPNet, MiniLM, and BioLORD-2023) to estimate variable similarity based on variable labels and derivation rules. A lexical matching method using fuzzy matching was included as a baseline model. In addition, we developed an ensemble-learning method, using the Random Forest (RF) model, to integrate individual NLP methods. RF was trained and evaluated on 50 trials. Each trial had a random split (4:1) of training and test sets, with the model’s hyperparameters optimized through cross-validation on the training set. For each EU variable, 1322 candidate JP variables were ranked based on NLP-derived similarity scores or RF’s probability scores, denoting their likelihood to match the EU variable. Ranking performance was measured by top-*n* hit ratio (HR-*n*) and mean reciprocal rank (MRR).

**Results:**

E5 performed best among individual methods, achieving 0.898 HR-30 and 0.700 MRR. RF performed better than E5 on all metrics over 50 trials (*P* < 0.001) and achieved an average HR-30 of 0.986 and MRR of 0.744. LLM-derived features contributed most to RF’s performance. One major cause of errors in automatic variable matching was ambiguous variable definitions.

**Conclusion:**

NLP techniques (especially LLMs), combined with ensemble learning, hold great potential in automating variable matching and accelerating biomedical data harmonization.

## Introduction

Epidemiology and machine learning studies in the biomedical domain often require large, diverse samples to produce unbiased results and improve the generalizability of findings [1,2]. However, such comprehensive data are rarely found in a single study. Instead, many datasets are generated by individual studies and shared via public platforms or data repositories [3]. Data sharing has become widely adopted in research communities and is now often mandated by funding agencies [4,5]. To effectively utilize the shared datasets, data harmonization is typically employed or required [6–8].

Data harmonization refers to the process of combining data from multiple resources to achieve maximum compatibility [6]. Strategies for data harmonization can be broadly categorized into two types: the stringent approach and the flexible approach [9]. The stringent approach uses the same measurements and data collection protocols across studies. Datasets from these studies share the same variables and can be harmonized through data merging directly [10]. In practice, implementation of this strategy is often challenging and typically confined to specific projects, due to the absence of widely accepted common data elements (i.e., standardized data definitions) and low adoption rates across individual studies [11]. In addition, the availability of numerous historical datasets necessitates effective harmonization methods that are both flexible and robust to maximize their usability [8]. The flexible approach does not require studies to use identical variables; instead, it uses analytical methods to transform matched variables into a common data model [12]. For example, the flexible prospective harmonization method requires researchers to agree on compatible data collection tools and protocols before the studies begin [13], while the flexible retrospective harmonization method can be applied to data from existing similar studies without prior collaboration or agreement among these studies [14–17].

All flexible retrospective harmonization methods share a common early step: identifying variables that can be merged or mapped across studies (which we call variable matching) [8,18,19]. Current data harmonization practices in the biomedical domain tackle this problem in manual and labor-intensive ways, where cohort experts identify relevant variables, assess their compatibility (based on variable definitions and data format), and estimate the level of difficulty of harmonization [8,18,19]. Even the initial step of identifying relevant variables across studies can be time-consuming, especially when datasets include numerous variables and use diverse naming conventions [16,17,19]. Approaches that automatically match variables across studies can reduce manual efforts and accelerate the data harmonization process. Research in this area has been limited, with methods primarily focusing on lexical matching and ontology-based semantic expansion and matching (e.g., using concepts and relations defined in ontologies to expand variables to be matched) [20–23] and keyword-based matching [24].

This study aimed to develop and evaluate a new variable matching approach that leverages large language models (LLMs) for variable matching to reduce human efforts and accelerate the data harmonization process. Using variables from two large cohort studies on Alzheimer’s Disease (AD) [25,26], we showed that the LLM-based methods outperformed the fuzzy matching method substantially in variable matching. In addition, we demonstrated that a tree-based ensemble classifier, which combined features from individual variable matching methods—including fuzzy matching and LLMs—significantly outperformed the best individual LLM method across all evaluation metrics over 50 trials. To our knowledge, this is the first study that demonstrates the great potential of applying LLMs and ensemble learning to support biomedical data harmonization.

## Materials and methods

### Approach overview

**Fig 1:**
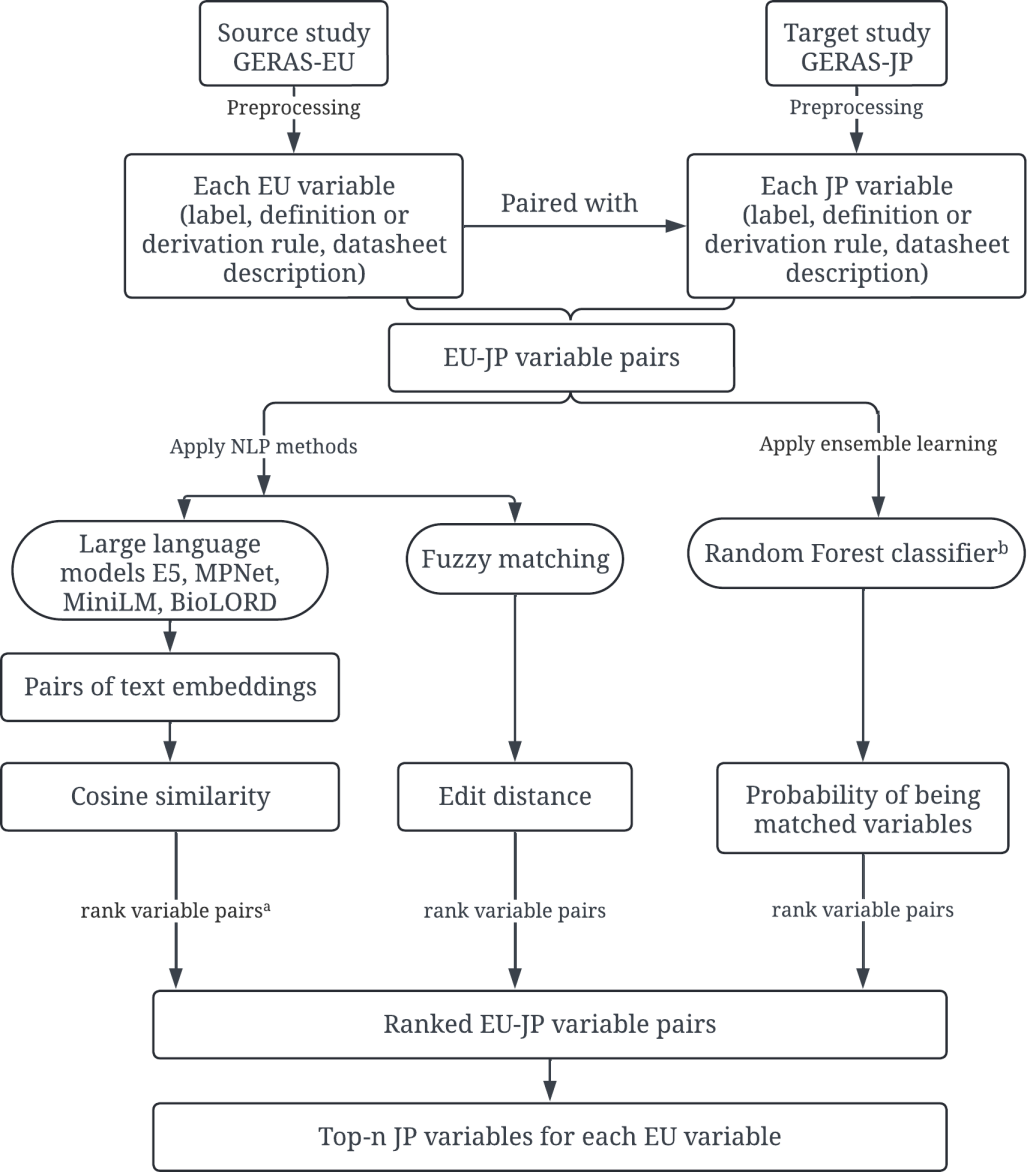
Overview of the automated variable matching approach. ^a^Each large language model (LLM) was applied to rank EU-JP variable pairs separately. b The features used by the Random Forest classifier included cosine similarity scores generated by LLMs, edit distance scores generated by fuzzy matching, and other features derived from the data dictionary (detailed in section Class label and machine learning features).

We treated the variable matching task as a ranking problem (Fig 1). For each source variable (i.e., a variable from GERAS-EU), we ranked candidate target variables (e.g., variables from GERAS-JP) based on their similarities to the source variable. The similarity between two variables was estimated by individual natural language processing (NLP) methods and ensemble learning, using information extracted from variable labels and other related sources (e.g., definitions or derivation rules of the variables, descriptions of data sheets containing the variables). Fig 1 provides an overview of our approach. This study was conducted as a secondary data analysis of deidentified data made available through the AD Data Initiative’s AD workbench and was exempt from the Institutional Review Board (IRB) review.

### Study setting

We mapped variables between the GERAS-EU [25] and GERAS-JP [26] studies, which were accessed through the AD Data Initiative’s AD Workbench—a secure, cloud-based data sharing and analytics environment to facilitate open data access and collaboration for AD-related research globally. The GERAS-EU study is an observational study examining the societal costs associated with AD in three European countries: France, Germany, and the United Kingdom [25]. This study recruited 1,532 participants with probable AD between October 1, 2010 and September 31, 2011 and collected data during the baseline visit and the follow-up visits every 6 months over 18 to 36 months. A variety of data elements were collected, such as demographics, medical history, cognitive function (e.g., Mini-mental state examination [MMSE] [27], the Alzheimer’s Disease Assessment Scale–Cognitive Subscale [28]), daily activities (Activities of Daily Living Inventory [29]), resource utilization (e.g., Resource Utilization in Dementia survey [30]), and quality of life (e.g., EuroQol-5 Dimension surveys [31]). The second study, GERAS-JP, utilized a similar study protocol to investigate the societal costs associated with AD in Japan [26,32]. It enrolls 553 participants with probable AD between November 2016 and December 2017 in Japan. Although GERAS-JP and GERAS-EU collected similar data, they recorded and formatted their data in different ways. For example, as shown in Table 1 (see Table A in S1 Text for more examples), the variable recording the participant’s body mass index was named “BMIB” (labeled as “Body Mass Index (BMI) at Baseline”) in GERAS-JP and “VSBLVTR_BMI” (labeled as “Vital Sign Result Numeric BMI baseline”) in GERAS-EU,

**Table 1:** Examples for variable names, labels, and data sheet descriptions^a^.

EU Variable Name	EU Variable Label	EU Data Sheet Description	JP Variable Name	JP Variable Label	JP Data Sheet Description
**Demographics**					
SEXLNM	Sex LNM	Demographics Relationship	SEX	Sex	Subject Level Analysis Dataset
**Anthropometric**					
VSBLVTR_BMI	Vital Sign Result Numeric BMI baseline	Vitals	BMIB	Body Mass Index (BMI) at Baseline	Subject Level Analysis Dataset
**Diagnosis/Treatment**					
DISCDLNM_Hypertension	Disease Code SNM: Hypertension	Medical History – Comorbidities	CCC09	Hypertension Diagnosis	Caregiver Comorbidities per visit
**Questionnaire**					
MMSERN_MMSES8	MMSE Item Result Numeric MMSES8 Correct response to orientation to place what is the city/town	Mini-Mental State Exam	MMSE_Q8	What is the city/town?	Mini-Mental State Examination (MMSE) per visit
**Time to event**					
TTERN_TTD	Time to Event Result Numeric (Time) TTD: Time to Death	Time to Event	TTDEATH	Time to Death (Months)	Subject Level Analysis Dataset
**Cost**					
COSPR1RN	Cost Primary Analysis 1 Item Result Numeric	Cost Caregiver Indirect Nonmedical	COST_INC_OP_C24_SUM	Caregiver Indirect Non-Medical Cost from Baseline up to the Visit: Opportunity Cost Approach, Supervision Time Not Included, Average Hours p.d. capped on 24	Total Cost up to the visit per visit

^a^Each row in the table is two matched variables from GERAS-EU and GERAS-JP studies

### Data preprocessing and information used for variable matching

We automatically transformed the data from long format to wide format before matching variables, if a variable was represented in different formats in the two studies. For example, MMSE scores in GERAS-EU were stored in long format, where the variable “MMSEQSNUM” recorded the MMSE question number and the variable “MMSERN” recorded the corresponding scores. In contrast, MMSE scores in GERAS-JP were stored in wide format, with variables such as “MMSE_Q1” and “MMSE_Q2” recording scores for each MMSE question separately. We transformed “MMSEQSNUM” and “MMSERN” in the GERAS-EU data jointly into wide format and assigned variable labels to the transformed variables accordingly. The same process was applied to all questionnaire-related variables originally represented in long format. Both studies stored longitudinally measured variables in the same long format by using a variable “VISITNUM” to index each visit. Because the time-intervals between visits were identical (every six months) for both studies, time-dependent matching can be solved by aligning the visit number. Therefore, we did not perform the long-to-wide transformation for these variables.

#### Variable labels and data sheet description.

Our approach matched variables based on information from data dictionaries, such as variable labels and data sheet descriptions. Table 1 provides examples of matched variables from the GERAS-EU and GERAS-JP studies (see Table A in S1 Text for more examples). Variable labels provide concise descriptions of the corresponding variable names. Both studies organized their variables in separate data sheets, with each data sheet representing a specific type of variables (e.g., demographic variables) or variables derived from a specific survey. Each data sheet was accompanied by a short description in the data dictionaries. For example, the GERAS-JP study kept variables associated with the MMSE test in a data sheet with the description “Mini-Mental State Examination (MMSE) per visit”. In this data sheet, the variable “MMSE_Q8” recorded the response to the eighth question in the MMSE [27].

#### Key word extraction from derivation rules.

The data dictionaries of both GERAS-EU and GERAS-JP studies also contain a field called variable definition or derivation rule (see Table B in S1 Text for examples). This field provides information about how the variables were defined or derived, such as the values of categorical variables and the derivation rules of variables. Derivation rules provide additional information beyond variable labels and data sheet descriptions, which can be valuable for variable matching. However, not all variables have derivation rules. In addition, there is significant variation in the length and content of derivation rules. Therefore, we utilized information from derivation rules exclusively for ensemble learning.

We incorporated information from derivation rules into variable matching as follows. First, if a derivation rule contained more than 20 words, KeyBERT [33] was employed to extract key words of up to 15 words to represent the entire text. KeyBERT utilizes BERT (Bidirectional Encoder Representation from Transformer) [34] embeddings to identify sub-phrases in a text that are most semantically similar to the original text. If the derivation rule contained 20 or fewer words, the entire text was retained. Second, for each variable, we concatenated the variable label with the derivation rule (or key words extracted from the derivation rule) to create input for the individual NLP methods. This treatment ensured that variables without derivation rules had non-empty input for NLP.

### Natural language processing methods for variable matching

We evaluated two types of NLP methods: LLM-based and fuzzy matching.

#### Large language model-based methods.

We evaluated four LLMs in variable matching: E5, MPNet, MiniLM, and BioLORD-2023.

The E5 (**E**mb**E**ddings from bidir**E**ctional **E**ncoder R**E**presentations) is a text embedding model that enhanced its training process through weakly supervised contrastive pre-training [35]. The key idea of E5’s contrastive pre-training is optimizing text embeddings so that they will bring relevant unlabeled text pairs closer together and push irrelevant text pairs further apart within the vector space of embeddings [36]. In E5, relevant text pairs were sourced from diverse platforms, including Reddit (posts and comments), Stack Exchange (questions and upvoted answers), English Wikipedia (entity names and passages), scientific papers (titles and abstracts), and Common Crawl web (titles and passages) and selected via a consistency-based data filtering technique [35]. These relevant text pairs served as positive examples; text from different relevant pairs formed irrelevant text pairs (i.e., negative examples). During pre-training, E5 enhanced existing LLMs, e.g., the BERT models by leveraging the large amount of newly collected text pairs and contrastive learning. The model was then fine-tuned using three labeled datasets: NLI (Natural Language Inference), MS-MARCO passage ranking dataset [37], and NQ (Natural Questions) [38] datasets. E5 outperformed existing embedding models on both BEIR [39] and MTEB [40] benchmark datasets that were used to evaluate a variety of text embedding tasks. This study used the E5_large model, which is built on the large BERT model Bert-large-uncased-whole-word-masking model. The E5_large_v2 model was chosen due to its superior performance on benchmark datasets, particularly in the semantic textual similarity (STS) tasks [35].

In addition, we evaluated two LLMs developed using the Sentence Transformers (also called SBERT) framework [41]. Built on Siamese and triplet networks and contrastive learning techniques, SBERT aims to generate semantically meaningful embeddings from sentences or short paragraphs while achieving higher computational efficiency than BERT [41]. SBERT incorporates pre-trained LLMs into their low-level building blocks. We evaluated two LLMs, MPNet and MiniLM, incorporated into SBERT [42].

The MPNet (masked and permuted language modeling) model unifies mask language modeling from BERT [34] and permuted language modeling from XLNet [43]. In addition, MPNet utilizes auxiliary position information (i.e., the tokens’ positions in the original, non-permutated input sentence) to improve the consistency of the model’s input representations between pre-training and fine-tuning [43]. The All_MPNet_base model was fine-tuned from the MPNet-base model using contrastive learning on over 1 billion text pairs sourced from diverse datasets (e.g., Stack-Exchange, MS-MARCO, NQ) [42]. Among all SBERT models, the All_MPNet_base model has demonstrated superior performance on the Sentence Embedding task (14 datasets) and the Semantic Search task (6 datasets) [42].

The MiniLM model employs a specific knowledge distillation technique, deep self-attention distillation, to compress large transformer-based models [44,45]. Knowledge distillation compresses a large, complex model (teacher model) into a smaller, simpler model (student model) with fewer parameters by minimizing the difference between intermediate features of the two models (e.g., self-attention distributions in Transformer models). It has been shown that the student model obtained through using this technique can maintain similar test accuracy as the teacher model across tasks such as image and speech recognition [46]. MiniLM employs distillation on the self-attention module of the final transformer layer of the BERT base model, resulting in a 12-layer student model [45,47]. This reduction in parameters enhances fine-tuning efficiency. In this study, we used All_MiniLM_L12 model [41,42]. Initialized from the pre-trained MiniLM (microsoft/MiniLM-L12-H384-uncased) model, All_MiniLM_L12 was fine-tuned with a contrastive objective using a dataset of 1 billion text pairs, including NQ and SQuAD2.0 [48]. This model is selected due to its comparable performance in sentence embedding and semantic search tasks when compared with the All_mpnet_base_v2 model [42].

We also evaluated BioLORD-2023, an LLM that leveraged the Unified Medical Language System (UMLS) knowledge graph to improve the generation of embeddings for text containing medical concepts [49]. The BioLORD-2023 model is based on the MPNet model (as described previously) [41] and leverages the LORD (**L**earning **O**ntological **R**epresentations from **D**efinitions) strategy [50,51] to further refine model training to enhance semantic representations of biomedical text. The training process of BioLORD-2023 involved three phases: contrastive learning, self-distillation, and weight-averaging. The contrastive learning phase implemented the LORD strategy [51]. Specifically, the model was trained on paired medical concept names and definitions to learn embeddings, ensuring that each concept’s name was close to its definition(s) while remaining distant from definitions of other concepts in the embedding space. The self-distillation phase aimed to mitigate performance degradation in measuring general-purpose semantic similarities, which arose due to intensive contrastive learning on medical concept names and definitions—a phenomenon observed in the previous BioLORD-2022 model [51]. In this phase, the knowledge acquired by the contrastive model (i.e., the model derived from the contrastive learning phase) was distilled into the base model in a supervised manner. Specifically, the concept embeddings and definition embeddings learned by the contrastive model were first averaged and then reduced to 64-dimensional vectors through principal component analysis. These reduced embeddings served as the learning objective for the base model, which was trained to predict them through a randomly initialized linear projection layer. Due to the random initialization of this projection layer, the training process produced multiple slightly different fine-tuned self-distillation models. To enhance robustness, the weight-averaging phase applied a strategy called “model soup” [52] to average the parameters of the fine-tuned self-distillation models to derive a single, ensembled model. BioLORD-2023 leveraged two biomedical knowledge graphs (ontologies)—the UMLS [53] and the Systematized Nomenclature of Medicine-Clinical Terms (SNOMED-CT) [54]—to enhance model training. Biomedical concepts and their definitions from the broad-coverage UMLS were used for contrastive learning. The training data were further augmented by: (1) extending concept definitions using template-based descriptions sampled from UMLS relations (e.g., is-a, used to treat, is the synonym of) and (2) incorporating 40,000 definitions from the Automatic Glossary of Clinical Terminology (AGCT) [55], which were automatically generated using the GPT-3.5 model and the SNOMED-CT ontology. BioLORD-2023 outperformed two BERT-based models (which incorporated knowledge from UMLS or biomedical literature via domain-specific pre-training or fine-tuning) and BioLORD-2022 (which incorporated knowledge from UMLS via contrastive learning) on both general-domain and biomedical-domain STS tasks [49].

#### Fuzzy matching.

Fuzzy matching methods utilize dynamic programming and edit distance [56] (e.g., Levenshtein distance) to assess lexical similarities between text strings. Edit distance measures the number of operations needed to transform one text string into another [56]. We computed the edit distance between the label of a GERAS-EU variable and the label of each GERAS-JP variable. Variables with a smaller edit distance were considered similar. We implemented the fuzzy matching method by using the Python package RapidFuzz [57], which incorporates a variety of edit distance scoring functions originally developed in the Fuzzywuzzy Package [58]. Prior to applying fuzzy matching, we preprocessed the variable labels by removing punctuations and stop words, converting all letters to lowercase, and stemming the words. We compared the performance of various fuzzy matching scoring functions implemented in the RapidFuzz package in a preliminary experiment and selected the top-performing function, the token-set-ratio function, for this study. The token-set-ratio function is an extension of the token-sort-ratio function. The token-sort-ratio function tokenizes the preprocessed strings, sorts the tokens alphabetically, and computes the Levenshtein distance between the sorted strings. The token-set-ratio function eliminates duplicate tokens within each sorted string before comparison.

### Ensemble learning for variable matching

To further improve variable matching performance, we employed the Random Forest classification algorithm to integrate the outputs from both LLM-based methods and the fuzzy matching method.

#### Random forest classifier.

**Fig 2:**
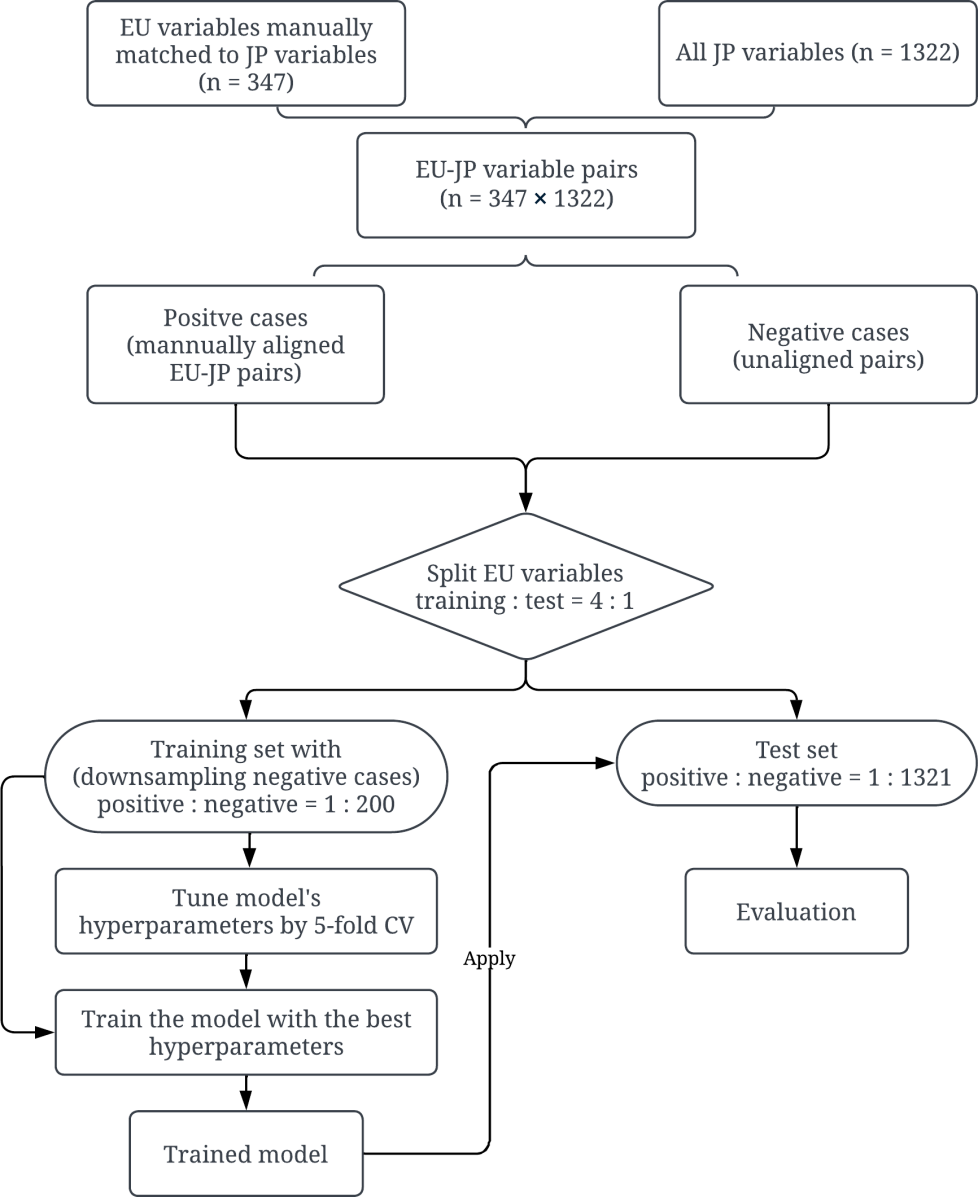
Training and evaluation of Random Forest (RF) classifier in a single trial^a^. ^a^ The Random Forest classifier was trained and evaluated in 50 trials, with each trial having a different random split of the training and test datasets. Each test set had slightly varying ratios of positive (i.e., matched EU-JP variable pairs) to negative (i.e., unmatched EU-JP variable pairs) cases because some EU variables were manually aligned to multiple JP variables (see details in sub-sections Training and test datasets and Model comparison).

The Random Forest classifier is a supervised machine learning model that utilizes an ensemble of decision tree classifiers to generate predictions. The classifier takes features associated with a pair of EU-JP variables as its input and outputs a class label (1: matched variables, 0: unmatched variables) and the associated probabilities. The training of the Random Forest classifier involves the creation of an ensemble of decision trees that classify the input from the training data. Each tree in the random forest builds on a subset of training instances randomly sampled from the complete training set without replacement, as well as a subset of features randomly selected from the entire feature set [59]. Introducing randomness enhances model robustness and helps mitigate overfitting. The construction of a decision tree involves splitting nodes iteratively from top to bottom. Each node is split based on a specific machine learning feature, with the feature and the corresponding splitting rule determined by criteria such as Gini impurity and information gain. For continuous features, the split rule may check whether the feature value is within a certain range. For categorical features, it may check whether the feature value is equal to a specific category. The goal is to achieve the greatest reduction in impurity or the largest increase in information gain with each split. The node-splitting process continues until certain termination criteria are met, such as reaching the maximum tree depth, the minimum number of samples required for a split, or the minimum decrease in impurity. When applying a trained Random Forest classifier to a new data instance, each decision tree that makes up the classifier is applied to the data instance respectively. At each node of a decision tree, the corresponding split rule will direct the data instance to a certain branch (i.e., a specific child node) under that node. After traversing several nodes in the tree, the data instance will reach a leaf node and receive its classification label (which is the label of the majority training instances that reach the same leaf node during model training). A final classification label is determined based on the aggregated voting results from all decision trees. In addition, the classifier will output a probability indicating how likely the data instance (in this case, a pair of variables) is positive, i.e., represents a match. These probabilities were then used to rank the candidate JP variables for each EU variable. Fig 2 provides an overview of the training and test procedures of the Random Forest model.

#### Training and test datasets.

The datasets utilized in this study were created in the following way.

First, we manually matched a subset of variables from GERAS-EU and GERAS-JP studies, which served as the ground truth variable pairs. Multiple steps were taken to ensure accurate matching. At the first step, EU variables were assigned to five co-authors, who had training and work experiences in epidemiology, statistics, or bioinformatics, for preliminary matching with corresponding JP variables. The variables were matched based on both variable definitions (i.e., derivation rules) and variable values. Challenging cases were discussed within the research team, including a senior co-author with expertise in epidemiology and health informatics. We categorized the matching results into three groups: no match, single match, and multiple matches. For example, the variable “LVLOCLNM” (which records the living area, urban or rural, of the participant) in the GERAS-EU study was matched with multiple variables such as “C_LIVLOCCD” and “LIVLOC” in the GERAS-JP study, because it was included in multiple data sheets under different variable names in GERAS-JP. At the second step, the five co-authors validated and corrected matching results from the first step, with each assigned a different subset of variables. The validation results and corrections (along with a justification for any corrections) were documented. In the last step, one co-author reviewed all corrections to ensure their validity. Through this 3-step procedure, 438 pairs of EU-JP variables were manually matched, which included 347 unique EU variables (68 of which have multiple true alignments).

Second, we created the training and test datasets in two steps (Fig 2). First, we treated the 347 unique EU variables as source variables that need to be aligned with the target variable(s) in the GERAS-JP study and randomly divided these variables into training and test (4:1) sets. Each EU source variable had 1322 JP candidate variables to match, which constituted 1322 variable pairs. These variable pairs contained only one or few positive instances (matched EU-JP pairs) but many negative instances (unmatched EU-JP pairs). Second, we down-sampled the negative instances to mitigate the adverse effects of data imbalance on model training. Specifically, for the EU source variable in each positive instance, we randomly selected 200 JP variables that did not match this EU variable to form 200 EU-JP variable pairs as negative training instances. In the test set, however, we included all the negative instances. This resulted in about 351 positive instances (associated with 277 unique EU variables) and 70,200 negative instances in the training set and 87 positive instances (associated with 70 unique EU variables) and 92,453 negative instances in the test set in each trial of the machine learning experiment (detailed in the section Experimental settings).

#### Class label and machine learning features.

The class label is binary-valued, with 1 indicating manually matched EU-JP variable pairs and 0 indicating unmatched EU-JP variable pairs. Features utilized by the Random Forest model came from two sources: similarity scores generated by five individual NLP methods (E5, MPNet, MiniLM, BioLORD-2023, and fuzzy matching) and other information extracted from data dictionaries. Each NLP method generated three similarity scores for an EU-JP variable pair by using (1) data labels; (2) data sheet descriptions; and (3) data labels and key words extracted from derivation rules as the respective input. For example, the EU variable “DIAGDT” (representing “Disease diagnosis date”) and the JP variable “ADDIADT” received three similarity scores from the E5 model calculated based on variable labels (0.98), data sheet descriptions (0.76), and data labels plus key words from derivation rules (0.90) respectively. Other features include the length of variable labels, the length and the absence of derivation rules for both the EU and JP variables. For example, for the EU variable “DIAGDT”, we had three features: 3 for the length of the variable label measured in words, 30 for the length of the derivation rule in words, and “False” for the absence of derivation rules (see Table B in S1 Text). In total, 15 similarity scores (three generated by each NLP method) and 6 additional features (3 for each variable in the variable pair) were used as machine learning features.

#### Model development.

In this study, we utilized the Random Forest classifier from the Scikit-learn package [60]. We developed the model using the training set and tuned the following hyperparameters through 5-fold cross validation and grid search: (1) the number of trees in the forest; (2) the maximum depth of each tree; (3) the criteria used to assess the quality of a tree node split; (4) the minimum number of samples required for each split; and (5) the number of features considered at each splitting. Other hyperparameters were set to their default values. We used the hit ratio (HR) at top 30 and mean reciprocal rank (MRR; see section Evaluation metrics) to select the best hyperparameters.

### Evaluation metrics

For each EU variable, each matching method will generate a ranked list of all candidate JP variables. A high-performance method is expected to place the manually matched JP variable(s) at the top of this list.

We evaluated the performance of the matching methods by two metrics: HR and MRR. Both HR and MRR are commonly used to evaluate recommendation or ranking algorithms [61,62]. As shown in equation 1, HR is defined as the total number of hits appearing in the top-*n* ranked items ($V_{hit}^{n}$) divided by the total number of search queries ($V_{all}$).


$$HR=V_{hit}^{n}/V_{all}$$ (1)

A “hit” denotes that the user-selected item is among the top-*n* ranked items. In our case, a “hit” means, for an EU variable, the manually matched JP variable is ranked within the top-*n* list of JP variables identified by the matching method. $V_{hit}^{n}$ denotes the total number of hits in the top-*n* lists for all EU variables in a dataset. $V_{all}$ denotes the total number of EU variables in the dataset.

For each user query *v*, the reciprocal rank RR(*v*) is the inverse of the rank of the first relevant item (equation 2). The MRR is the averaged reciprocal rank across all queries (equation 3).


$$RR(v)=1/R_{hit}$$ (2)

$$\begin{array}{c} MRR=\sum {\mathrm{\backslash nolimits}}_{v=1}^{all}RR(v)/V_{all} \end{array}$$ (3)

In our case, $R_{hit}$ in equation 2 denotes the highest (smallest) rank of the JP variable(s) that were manually matched to an EU variable *v*. MRR is the averaged reciprocal rank across all EU variables in a dataset.

Ties in ranking were resolved by using the median rank when calculating HR and MRR. For example, if three JP variables have identical similarity scores when matched to an EU variable and occupy positions 4–6 in the ranked list, they will all be assigned a rank of 5.

### Experimental settings

#### Model comparison.

We first compared the performance of the five individual NLP methods on the whole dataset. We then compared the performance of the Random Forest classifier and the best NLP method on the test sets from 50 trials. The 50 trials were conducted by randomly splitting training and test datasets 50 times (see section Training and test datasets). We used paired *t*-tests to compare the performance of the two models across the 50 trials, with the null hypothesis stating that there is no significant difference in performance metrics between these two methods. Five metrics were used to evaluate model performance, including the top 30, 20, 10, and 5 HR (HR-30, HR-20, HR-10, HR-5) and MRR.

#### Feature importance analysis.

To understand which features contributed most to the performance of the Random Forest model, we estimated feature importance using data from the 50 trials and the permutation importance method [63]. For each feature in a trained model, permutation importance estimates its contribution by randomly shuffling the feature’s values in a held-out dataset and measuring the subsequent decline in model performance on this dataset. In this study, we estimated feature importance by averaging the model’s performance decline over the test sets from the 50 trials, measured by HR-5, HR-10, and MRR. We selected these three metrics because they were more sensitive to permutation on individual features in the Random Forest classifier compared to HR-20 and HR-30.

We compared feature importance using two methods. In the first method, we ranked features using their mean importance scores over 50 trials. In the second method, we first ranked the features within each trial based on their importance scores and then compared their average ranks across the 50 trials.

In addition, we conducted feature ablation analysis to assess whether removing a specific type (i.e., subset) of features from the Random Forest model would affect the model performance. We categorized the features into three types: LLM-derived features, fuzzy matching-derived features, and other features. We measured the average decline in model performance after removing each type of feature over 50 trials.

### Error analysis

To understand the limitations of our variable matching approach, we manually checked the manually matched EU-JP variable pairs that were ranked low (below top-30) by the best NLP method and the Random Forest model and summarized the error patterns.

## Results

### Descriptive statistics

The dataset used in this study included 347 GERAS-EU variables and 1322 GERAS-JP variables. As shown in Table 2, compared with the JP variables, the EU variables have longer labels (11.3 vs. 8.5 words), shorter data sheet descriptions (3.2 vs. 5.5 words), and shorter derivation rules (14.9 vs. 26.4 words).

**Table 2:** Characteristics of variable labels, data sheet descriptions, and derivation rules of EU and JP variables.

	EU (n = 347)		JP (n = 1322)	
	Mean (std)^a^	Median [Q1, Q3]^a^	Mean (std)^a^	Median [Q1, Q3]^a^
Number of words in label	11.3 (8.2)	9 [4,18]	8.5 (5.6)	7 [5,10]
Number of words in data sheet description	3.2 (1.2)	3 [2,4]	5.5 (1.8)	5 [4,7]
Number of words in derivation rule	14.9 (154.5)	0 [0,2]	26.4 (38.9)	11 [4,33]

^a^Q1: First quantile; Q3: Third quantile; std: standard deviation.

Table A in S2 Text provided descriptive statistics for features used in trial 1 of the machine learning experiment. Other trials showed similar patterns in feature value distributions. The NLP-derived features (i.e., similarity scores estimated by the individual NLP methods) showed similar distributions in both the training and test sets. A higher score or feature value indicates a greater similarity between the EU and JP variables.

Other features used in the Random Forest model were summarized in Table B in S2 Text. The GERAS-EU study exhibits a shorter median and mean keyword length (medium length: 9 words, mean length: 8.8 words) in non-empty derivation rules than the GERAS-JP study (medium length: 15 words, mean length: 11.4 words). The EU variables had longer labels (medium length: 9 words, mean length: 11.3 words) than the JP variables (medium length: 7 words, mean length: 8.5 words). About 72.6% of EU variables missed the derivation rules, while only 11.5% of JP variables missed the derivation rules.

### Comparison of individual NLP methods

As shown in Table 3, the E5 model achieved the highest performance across all evaluation metrics, including an HR-30 of 0.898 and an MRR of 0.700. The BioLORD-2023 model achieved the second-best performance across all metrics, followed by the MiniLM and MPNet models. The fuzzy matching method exhibited the lowest performance in most metrics but outperformed the MPNet model in HR-5 and MRR.

**Table 3:** Performance of individual NLP methods in variable matching^a, b^.

Models	HR-5	HR-10	HR-15	HR-20	HR-30	MRR
E5	**0.798**	**0.861**	**0.886**	**0.889**	**0.898**	**0.700**
MiniLM	0.662	0.730	0.778	0.795	0.841	0.551
MPNet	0.608	0.705	0.733	0.761	0.798	0.511
BioLORD	0.753	0.804	0.838	0.858	0.884	0.674
Fuzzy matching	0.616	0.702	0.733	0.744	0.776	0.549

^a^The individual models were evaluated on 347 EU variables that were manually matched with JP variables.

^b^HR-30: Top 30 hit ratio; HR-20: Top 20 hit ratio; HR-10: Top 10 hit ratio; HR-5: Top 5 hit ratio; MRR: Mean reciprocal rank; E5: E5_Large_V2 model; MiniLM: All_MiniLM_L12 model; MPNet: All_MPNet_base_V2 model; BioLORD: BioLORD-2023 model; Fuzzy matching: the token set ratio method. All methods used only variable labels to estimate variable similarities.

### Random Forest model versus E5

As shown in Table 4, the Random Forest model, optimized based on the HR-30 metric, outperformed the E5 model on all the evaluation metrics and achieved an HR-30 of 0.986 (std: 0.012) and an MRR of 0.744 (std: 0.036). Paired *t*-tests on the 50 trials indicated significant performance gains (*P* < 0.001 for all metrics). A separate Random Forest model, optimized based on MRR, exhibited similar performance across all metrics (see Table C in S2 Text).

**Table 4:** Random Forest and E5 model performance comparison.

Metric^a^	E5^b^ mean (standard deviation)	Random Forest^b^ mean (standard deviation)	Mean difference^b^ (Random Forest – E5), 95% confidence interval	*P* value
HR-30	0.911 (0.029)	0.986 (0.012)	0.075 [0.067, 0.084]	<0.001*
HR-20	0.905 (0.028)	0.975 (0.017)	0.070 [0.062, 0.078]	<0.001*
HR-10	0.871 (0.034)	0.929 (0.027)	0.058 [0.048, 0.068]	<0.001*
HR-5	0.803 (0.040)	0.872 (0.038)	0.069 [0.059, 0.079]	<0.001*
MRR	0.659 (0.044)	0.744 (0.036)	0.086 [0.076, 0.095]	<0.001*

^a^HR-30: Top 30 hit ratio; HR-20: Top 20 hit ratio; HR-10: Top 10 hit ratio; HR-5: Top 5 hit ratio; MRR: Mean reciprocal rank; E5: E5_large_V2 model.

^b^The performances of E5 and Random Forest compared over 50 trials, using the paired *t*-tests. The Random Forest model’s hyperparameters were optimized using the HR-30 metric.

### Feature analysis

As shown in Table 5, the features derived by applying the E5 model and the BioLORD-2023 model on variable labels (i.e., E5_on_label and BioLORD_on_label) contributed most to the performance of the Random Forest model, as indicated by all three evaluation metrics. Other features differed in their contributions measured by HR and MRR. Their contributions to HR-5 were all substantially lower compared to the two most important features. Features derived from applying MiniLM to variable labels (i.e., MiniLM_on_label), E5 to both variable labels and keywords extracted from the derivation rules (i.e., E5_on_label_key), and E5 to data sheet descriptions (i.e., E5_on_sheet) were among the top-ranked features for HR-10, with an average importance score of 0.010 or higher. In contrast, features derived from applying BioLORD-2023 to both variable labels and keywords extracted from the derivation rules (i.e., BioLORD_on_label_key), as well as applying MPNet and MiniLM to data sheet descriptions (i.e., MPNet_on_sheet and MiniLM_on_sheet) were important to MRR.

**Table 5:** Feature importance for the Random Forest model^a^.

	HR-5^b^		HR-10^b^		MRR^b^	
**Features** ^**c**^	**Average importance**	**Average rank**	**Average importance**	**Average rank**	**Average importance**	**Average rank**
**E5_on_label**	**0.036**	**2.66**	**0.034**	**2.86**	**0.041**	**2.58**
**BioLORD_on_label**	**0.034**	**2.64**	**0.029**	**3.46**	**0.037**	**2.80**
**MPNet_on_sheet**	0.008	7.38	0.007	9.26	**0.010**	**8.30**
**MiniLM_on_label**	0.006	10.60	**0.020**	**8.22**	0.005	12.28
**E5_on_label_key**	0.005	10.36	**0.014**	**7.52**	0.007	11.74
**BioLORD_on_label_key**	0.005	10.12	0.010	9.14	**0.015**	**6.74**
**Fuzzy_on_sheet**	0.004	9.82	0.005	11.46	0.004	11.96
**E5_on_sheet**	0.004	9.40	**0.010**	**8.26**	0.008	8.94
**BioLORD_on_sheet**	0.003	10.04	0.009	8.34	0.008	9.18
**Derive_info_len_JP**	0.003	10.60	0.004	11.46	0.007	9.24
**Label_len_EU**	0.002	11.48	0.006	10.38	0.002	13.20
**MiniLM_on_sheet**	0.002	11.32	0.004	11.96	**0.010**	**8.40**
**Fuzzy_on_label**	0.001	11.82	0.003	12.48	0.008	9.72
**Label_len_JP**	0.000	12.16	0.005	11.20	0.003	12.62
**Derive_info_null_JP**	0.000	11.22	0.001	12.66	0.000	14.20
**Derive_info_null_EU**	−0.001	12.00	0.002	11.88	0.001	14.34
**MPNet_on_label_key**	−0.002	12.82	0.001	14.34	0.005	11.98
**Derive_info_len_EU**	−0.002	12.96	0.001	13.22	0.001	13.92
**Fuzzy_on_label_key**	−0.004	14.10	0.003	12.70	−0.004	15.22
**MPNet_on_label**	−0.006	14.84	0.002	13.06	−0.003	15.28
**MiniLM_on_label_key**	−0.008	15.42	0.001	13.82	−0.011	18.36

^a^NLP-derived features are similarity scores calculated by four large language models (E5, MPNet, MiniLM, BioLORD-2023) and fuzzy matching using variable labels, data sheet descriptions, and key words extracted from the derivation rules (detailed in section Class label and machine learning features). Top-ranked features with an average importance score of 0.010 or higher were bolded. The Random Forest model optimized based on the HR-30 metric was used in this experiment.

^b^HR-10: Top 10 hit ratio; HR-5: Top 5 hit ratio; MRR: Mean reciprocal rank

^c^“on_label” in a feature name indicates similarity scores based on variable labels; “on_sheet” indicates similarity scores based on data sheet descriptions; “on_label_key” indicates similarity scores based on the combination of variable labels and key words extracted from derivation rules; “Label_len” indicates the number of words in the variable label; “Derive_info_len” indicates the number of words in the derivation rule; “Derive_info_null” indicates whether the derivation rule is empty.

Feature ablation analysis (Table 6) showed that removing LLM-derived features or other types of features decreased the model’s performance significantly (*P* < 0.001 for most cases) while removing fuzzy matching features did not affect model performance except for HR-10 (*P* = 0.001).

**Table 6:** Feature ablation analysis^a^.

	Model 1: all features	Model 2: w/o LLM features		Model 3: w/o fuzzy matching features		Model 4: w/o other features	
		Δ (Model 1 – Model 2)^b^	*P* value	Δ (Model 1 – Model 3)^b^	*P* value	Δ (Model 1 – Model 4)^b^	*P* value
HR-30	0.986 [0.982, 0.990]	0.078 [0.069, 0.087]	<0.001*	0.001 [−0.002, 0.004]	0.47	0.001 [−0.003, 0.004]	0.62
HR-20	0.975 [0.970, 0.980]	0.105 [0.094, 0.116]	<0.001*	0.001 [−0.003, 0.005]	0.78	0.007 [0.003, 0.011]	<0.001*
HR-10	0.929 [0.922, 0.937]	0.119 [0.106, 0.131]	<0.001*	0.010 [0.004, 0.016]	0.001*	0.009 [0.004, 0.013]	<0.001*
HR-5	0.872 [0.861, 0.883]	0.157 [0.140, 0.174]	<0.001*	0.002 [−0.008, 0.004]	0.44	0.005 [0.000, 0.009]	0.048*
MRR	0.744 [0.734, 0.755]	0.172 [0.160, 0.184]	<0.001*	0.004 [−0.003, 0.010]	0.24	0.006 [0.002, 0.010]	0.01*

^a^HR-30: Top 30 hit ratio; HR-20: Top 20 hit ratio; HR-10: Top 10 hit ratio; HR-5: Top 5 hit ratio; MRR: Mean reciprocal rank. The Random Forest model optimized based on the HR-30 metric was used in this experiment.

^b^The performance decline of the partial model (i.e., model with features removed), relative to the full model, is summarized by the mean difference and its 95% confidence interval, calculated over 50 trials.

## Discussion

Variable matching is an early key step in flexible data harmonization. We evaluated the performance of NLP methods, including LLMs and fuzzy matching, on this task. In addition, we developed and evaluated a Random Forest-based ensemble learning method that leveraged the strengths of individual NLP methods. We found that the E5 LLM outperformed other individual NLP methods on variable matching. The Random Forest model showed significantly better performance than E5 on all metrics and achieved an HR-30 of 0.986 and an MRR of 0.744. These results suggest that NLP techniques (including LLMs), combined with ensemble learning, have great potential in automating variable matching, thus accelerating the data harmonization process. Below, we discuss our main findings in greater detail.

Data harmonization has been discussed within the context of utilizing data from multiple sources, which aims to combine datasets for effective use by resolving data heterogeneity at three levels: syntax (i.e., data format), structure (i.e., conceptual schema), and semantics (i.e., how the variables were measured, derived, and encoded) [6]. The advantages of data harmonization include increasing the statistical power of a study and enabling big data analytics [6,12,64], verifying findings across studies [1], and evaluating and reducing the bias of analyses using individual data sources [2,7]. Two strategies, merging and mapping, apply to the data harmonization process [6]. Merging involves the creation of a single global taxonomy or ontology for multiple datasets and then linking or mapping variables to the taxonomy [14,65]. Mapping, on the other hand, involves the creation of a set of rules to match variables across studies [6]. For example, Kamala et al. harmonized data from two pregnancy cohort studies by using the mapping approach, where they created a set of rules by considering construct measured, question asked, response options, measurement scale, time and frequency of measurement, and coding schema of variables, to classify variables into completely matching, partially matching (e.g., two variables measured the same construct but were measured or encoded in different ways), or no matching [12]. The mapping was conducted manually by reviewing documentation, consulting the research team of the original data source, and exploring variables in the dataset. Kamala’s team harmonized 20 variables from both cohorts and pointed out that this is a repetitive and iterative process [12]. In both mapping and merging approaches, matching variables between studies and the common data ontology, or across studies, can be time-consuming but remains a crucial step in data harmonization. Automated methods that facilitate variable matching, including the methods developed in this study, can potentially improve efficiency and, therefore, enable large-scale harmonization (e.g., harmonizing large datasets or many datasets).

In this study, we developed and tested new automated methods, leveraging LLMs and ensemble learning, to match variables across studies based on information provided in data dictionaries. Our approach relies on text comparison (e.g., comparing variable labels, data sheet descriptions, and derivation rules) and focuses on construct-level variable matching. Specifically, we considered both lexical similarity and semantic similarity of the text describing the variables [66]. The assessment of lexical similarity was motivated by the observation that some matched variables shared common technical terms in their definitions, such as variable labels or derivation rules. Fuzzy matching (approximate string matching [67]) focuses on measuring lexical similarity. The assessment of semantic similarity was motivated by the observation that matched variables could use different but semantically related words in their definitions. Text embedding is a widely used NLP technique for distributed text representation which can be used to measure semantic similarities between text [68]. Recent studies have shown the success of using sentence embeddings generated from BERT-based models to measure text semantic similarity [36,41]. LLMs, like BERT, KeyBERT, and RoBERTa, also showed high performance in measuring short-text semantic similarities [69]. Furthermore, incorporating information from biomedical ontologies (e.g., UMLS) or knowledge sources (e.g., the website of Mayo Clinic) into LLMs has been shown to enhance performance in downstream tasks such as question answering [70], generating synthetic electronic health records [71], and measuring semantic similarity between biomedical texts [49]. In this study, we treated variable matching as a short-text (a blend of medical concepts and ordinary text) similarity assessment task and utilized multiple pre-trained LLMs (which have been fine-tuned for either general or biomedical STS tasks) to measure the semantic similarity between variable definitions. The evaluation of individual NLP methods showed that LLMs outperformed fuzzy matching, suggesting that measuring semantic similarities was beneficial for variable matching. We also found that BioLORD-2023 outperformed its based model, MPNet, achieving absolute gains of 0.09 to 0.16 points across all evaluation metrics. This result demonstrates the benefits from incorporating knowledge graphs such as UMLS and SNOMED-CT in variable matching. It is worth noting that incorporating domain-specific knowledge into general-domain LLMs is a nontrivial task. Previous research has shown that fine-tuning an LLM originally designed for general-domain STS tasks through contrastive learning from biomedical text pairs may negatively impact the model’s ability to retain general knowledge [49]. BioLORD-2023 employed a self-distillation strategy to mitigate this negative effect. However, it still performed worse than the general-domain E5 model in variable matching. Future research in developing and comparing methods for integrating domain-specific knowledge into E5 may further advance the state of the art in variable matching by individual LLMs. In addition, methods inspired by retrieval-augmented generation (RAG) [72]-like techniques could potentially enhance LLMs for variable matching. For example, these methods could be used to retrieve text pairs (e.g., query-response pairs) containing medical terms from web posts to enrich the training data with more diverse and contextually relevant biomedical text pairs. Additionally, augmenting LLM inputs (e.g., variable labels and derivation rules in our case) with synonyms or concept relations extracted from biomedical knowledge graphs may further improve biomedical variable matching.

In addition, ensemble learning, which combines the strengths of multiple individual methods, is an effective approach for enhancing task performance. Our evaluation results showed that the Random Forest classifier consistently outperformed the best individual method (i.e., the E5 model). This aligns with prior studies demonstrating that ensemble-based systems are more effective than single-expert systems [73]. In our case, the single-expert system, such as the fuzzy matching method or the LLMs, provides similarity scores for two variables. Due to variations in algorithm design and model training methods, these single-expert systems may offer diverse perspectives on the nuances of similarities and differences between variable definitions. Furthermore, ensemble learning provides a flexible framework for incorporating different information sources (e.g., variable labels, data sheet descriptions, and variable derivation rules in our case), which is also called data fusion [73]. In this study, we used the Random Forest classifier to incorporate similarity scores measured by using different single-expert systems and information sources, as well as several additional features (e.g., lengths of variable labels), to classify each candidate GERAS-JP variable as matching or not matching a GERAS-EU variable. Results from feature importance analysis and feature ablation analysis showed that features derived by using LLMs contributed the most to the Random Forest model’s good performance. In addition, the feature importance analysis showed that the important features for a high hit ratio (which is sensitive to the quality of top-ranked variable pairs) and a high MRR (which measures the global or overall ranking quality) differed, although the E5-derived feature E5_on_label and BioLORD-derived feature BioLORD_on_label stood out as the most important for both metrics. The contribution of the fuzzy matching feature seemed negligible in feature ablation analysis, except for the top-10 hit ratio. A possible reason is that LLM features already capture sufficient information about both lexical and semantic similarities for matching variables.

Our error analysis revealed two major error patterns. First, the variable descriptions are sometimes ambiguous or lack specific details. For example, the E5 model and the Random Forest model failed to match the variable “MMSEBLVALTR” (labeled as “Baseline Value - TR Phase”) from the GERAS-EU study with the variable “MMSEB” (labeled as “Mini-Mental State Examination (MMSE) at Baseline”) from the GERAS-JP study. Both variables represent the MMSE performance at the baseline visit, but the label for the “MMSEBLVALTR” variable (which does not specify that this variable represents the baseline value of the MMSE test) is too ambiguous or vague to be useful for variable matching. Second, the variable labels from the two studies sometimes emphasized different perspectives. For example, the variable “MMSES34” was labeled as “Item result numeric: Correct response to writing” in the GERAS-EU study, and its counterpart in the GERAS-JP study, i.e., MMSE_Q34, was labeled as “Please write a sentence”. Both variables represent the evaluation result of item 34 in the MMSE questionnaire, but the variable label from the GERAS-EU study focuses on the evaluation result, whereas the label from the GERAS-JP study focuses on the evaluation item. In general, most error cases involved variable labels that lacked semantic and lexical similarities, making them challenging for the NLP methods to analyze effectively.

This study has limitations. We validated our approach on two studies that followed similar protocols to collect data and used data dictionaries with comparable formats. For example, both data dictionaries include data fields for variable name, variable label, data sheet description, and variable derivation rule. Additional work in data preprocessing is required when applying our methods to cases where data dictionaries differ substantially in format and structure across studies. However, the methodology underlying our approach is generic. By utilizing pre-trained LLMs and the LLM-derived text similarity scores as machine learning features, our approach can be applied to other settings (e.g., ontology-based retrospective data harmonization, searching variables of interest in existing datasets) where text descriptions of variables are available. Another limitation, common to all computational approaches, is the additional effort required to prepare input data when “computer-readable” documentation is unavailable for some studies included in a data harmonization project [19].

## Conclusion

Our NLP methods, which leveraged LLMs and ensemble learning, achieved promising results in the task of variable matching. Variable matching is an early key step in data harmonization, which often requires substantial human efforts. Our methods have great potential to reduce manual effort when text descriptions of variables are available for studies. In the future, we aim to refine and extend our methods to address scenarios with greater variation in data collection protocols across studies.

## Supporting information

S1 Text Data fields used by natural language processing methods to match variables. **Table A**. Examples for variable names, labels, and data sheet descriptions. **Table B**. Examples of KeyBERT extraction from derivation rules.
(PDF)

S2 Text Additional experimental results. **Table A**. NLP-derived features used by the Random Forest model. **Table B**. Other features used by the Random Forest model. **Table C.** Random Forest and E5 model performance comparison.
(PDF)

## Abbreviation:

AD: Alzheimer’s Disease
AGCT: automatic glossary of clinical terminology
BEIR: benchmarking-IR
BERT: bidirectional encoder representations from transformers
BMI: body mass index
E5: EmbEddings from bidirEctional Encoder rEpresentations
EU: European union
GERAS: a prospective observational study of costs and resource use in community Dwellers with Alzheimer’s Disease
HR: hit ratio
IRB,: Institutional Review Board;
JP: Japan
LLMs: large language models
LORD: learning ontological representations from definitions
MPNet: masked and permuted pre-training for language understanding
MiniLM: deep self-attention distillation for task-agnostic compression of pre-trained transformers
MRR: mean reciprocal rank
MMSE: the mini mental state examination
MTEB: massive text embedding benchmark
NLI: natural language inference
NQ: natural questions
NLP: natural language processing
RAG: retrieval-augmented generation
RF: random forest
STS: semantic textual similarity
SBERT: sentence-BERT
SNOMED-CT: systematized nomenclature of medicine-clinical terms
UMLS: unified medical language system
XLNet: generalized autoregressive pretraining for language understanding.
