# Supplementary material for: A natural language processing approach to support biomedical data harmonization: Leveraging large language models
Source: PLoS One. 2025 Jul 24;20(7):e0328262. doi: 10.1371/journal.pone.0328262 (PMC12289046; doi:10.1371/journal.pone.0328262)
Supplement: S1 Text — Data fields used by natural language processing methods to match variables. Table A. Examples for variable names, labels, and data sheet descriptions. Table B. Examples of KeyBERT extraction from derivation rules. (PDF) [file pone.0328262.s001.pdf]

S1 Text. Data fields used by natural language processing methods to match variables

Table A. Examples for variable names, labels, and data sheet descriptions [a]

EU Variable Name	EU Variable Label	EU Data Sheet Description	JP Variable Name	JP Variable Label	JP Data Sheet Description
**Demographics**					
LVLOCLNM	Living Location LNM	Living Arrangement	C_LIVLOCCD	Living Location Code	Dataset with all covariates
DMMARST	Marital status	Demographics Relationship	MRTLSTCD	Marital Status - Code	Subject Level Analysis Dataset
SEXLNM	Sex LNM	Demographics Relationship	SEX	Sex	Subject Level Analysis Dataset
**Anthropometric**					
VSBLVTR_BMI	Vital Sign Result Numeric BMI baseline	Vitals	BMIB	Body Mass Index (BMI) (kg/mÂ²) at Baseline	Subject Level Analysis Dataset
VSBLVTR_HGT	Vital Sign Result Numeric HGT baseline	Vitals	HGTCMB	Height (cm) at Baseline	Subject Level Analysis Dataset

VSBLVTR_WGT	Vital Sign Result Numeric WGT baseline	Vitals	WGTKGB	Weight (kg) at Baseline	Subject Level Analysis Dataset
**Diagnosis/Treatment**					
TRTSORT	Treatment Sort Order	Demographics Information	SEVGRPCD	AD Severity Group - Code	ADAS-Cog data per visit
DISCDLNM_Hypertension	Disease Code SNM: Hypertension	Medical History - Comorbidities	CCC09	Hypertension Diagnosis	Comorbidities per visit
DIAGDT	Disease Diagnosis Date	Medical History Disease Characteristics	ADDIADT	Alzheimer's Diagnosis Date	Subject Level Analysis Dataset
DEPRFLG	Depression Medication Flag	Solicited Treatment	PCC02	Depression Medication	Comorbidities per visit and over study
SDYTRTTERM	Study Treatment Dictionary Term	Study Treatment	ADTTERM	AD Treatment Name	All AD medication as recorded
SDYTRTSTDT	Study Treatment Start Date	Study Treatment	ADTSTDT	AD Treatment Start Date	All AD medication as recorded
CMNAME	Therapy Name	Concomitant and Previous Therapy	PHTTERM	Psychiatric/Hypnotic Medication Name	AD medication after Baseline
**Questionnaire**					

VISDT	Visit Date	Visit	VSDT	Visit Date	Visit dataset
MMSERN_MMSES8	MMSE Item Result Numeric MMSES8 Correct response to orientation to place what is the city town	Mini-Mental State Exam	MMSE_Q8	What is the city/town?	Mini-Mental State Examination (MMSE) per visit
MMSESEV	MMSE Severity	Mini-Mental State Exam	SEVGRPCDV	AD Severity Group at Visit - Code	Mini-Mental State Examination (MMSE) per visit
ADLASMDT	ADL Assessment Date	Activities of Daily Living Inventory	ADCSDT	Date of ADCS-ADL	ADAS-ADL data per visit
NPIBLVALTR	Baseline Value - TR Phase	Neuropsychiatric Inventory	BASE	Baseline Value	NPI data per visit
NPIRN_NPIS13	NPI Item Result Numeric NPIS13: Caregiver Distress: Delusions	Neuropsychiatric Inventory	NPIA_DSC	Caregiver Distress Score: Delusions Distress Score	NPI data per visit
EQ5RN_EQ5S2	EQ-5D Item Result Numeric EQ5S2: Self-Care	health-related quality of life	EQ5DC2	Caregiver: Self-care	EQ5D for caregivers per visit

RUDRN_RUDRN52A	RUD Item Result Numeric RUDRN52A: Please specify the principal reason for this change in living accommodation.	Resource Utilization in Dementia	LIVACCHGRSCD	Principal Reason for Changed in Living Accommodation since Last Visit - Code	RUD Patient Dataset per visit
ADASRN_ADASS6	ADAS Item Result Numeric ADASS6: Naming Objects and Fingers	Alzheimer's Disease Assessment Scale	ADAS_T04	ADAS-COG: Naming Objects and Fingers	ADAS-Cog data per visit
ADASRN_ADASS11	ADAS Item Result Numeric ADASS11: Word Recognition Task	Alzheimer's Disease Assessment Scale	ADAS_T07	ADAS-COG: Word Recognition	ADAS-Cog data per visit
**Time to event**					
TTERN_TTIN	Time to Event Result Numeric (Time) TTIN: Time to Institutionalization	Time to Event	TTINST	Time to Instituationalisation (Months)	Subject Level Analysis Dataset
TTERN_TTDS	Time to Event Result Numeric (Time) TTDS: Time to Discontinuation	Time to Event	TTDISC	Time to Discontinuation (Months)	Subject Level Analysis Dataset

TTERN_TTD	Time to Event Result Numeric (Time) TTD: Time to Death	Time to Event	TTDEATH	Time to Death (Months)	Subject Level Analysis Dataset
**Cost**					
COSTD	Cost Item Detail	Cost Caregiver Indirect Nonmedical	None	None	None
COSPR1RN	Cost Primary Analysis 1 Item Result Numeric	Cost Caregiver Indirect Nonmedical	COST_INC_OP_C24_SUM	Caregiver Indirect Non-Medical Cost from Baseline up to the Visit: Opportunity Cost Approach, Supervision Time Not Included, Average Hours p.d. capped on 24	Total Cost up to the visit per visit

[a] Each row is a matched variable pair between GERAS-J and GERAS-EU cohort.

Table B: Examples of KeyBERT extraction from derivation rules [a]

Variable Name	Variable Label	Derivation Rule	Derivation Rule key words	Label and key words
SEXLNM [b]	Sex LNM	Female / Male	Female / Male	Sex LNM, Female / Male
TRTSORT [b]	Treatment Sort Order	Treatment Group (1,2,3)	Treatment Group (1,2,3)	Treatment Sort Order, Treatment Group (1,2,3)
SDYTRTTERM [b]	Study Treatment Dictionary Term	Approved AD treatment Donepezil Galantamine Investigational product Memantine Rivastigmine Other	Approved AD treatment Donepezil Galantamine Investigational product Memantine Rivastigmine Other	Study Treatment Dictionary Term, Approved AD treatment Donepezil Galantamine Investigational product Memantine Rivastigmine Other
DISDUR [b]	Disease Duration	(ICDT-DIAGDT)/365.25	(ICDT-DIAGDT)/365.25	Disease Duration, (ICDT-DIAGDT)/365.25
CARGFLOWSNM [c]	Caregiver Flow SNM	Use across subject taking into account caregiver disposition up to Visit 7:<br>1 - 'Same caregiver'.<br>2 - '1st caregiver discontinued, no 2nd caregiver'.<br>3 - '1st caregiver discontinued; 2nd caregiver enrolled'.<br>4 - '1st caregiver discontinued; 2nd caregiver discontinued'.	Taking account caregiver disposition visit<br>Caregiver discontinued 2nd caregiver discontinued<br>Use subject taking account caregiver	Caregiver Flow SNM, Taking account caregiver disposition visit. Caregiver discontinued 2nd caregiver discontinued. Use subject taking account caregiver.

VSTESTCD [c]	Vital Sign Name	HGT for Height<br>WGT for<br>Weight<br>DIA for BP Diastolic Result<br>SYS for BP Systolic Result<br>HRT for Heart Rate Result<br>BMI for BMI	Result SYS BP Systolic result<br><br>Heart rate result BMI BMI<br><br>HGT Height WGT Weight DIA	Vital Sign Name, Result SYS BP Systolic result. Heart rate result BMI BMI. HGT Height WGT Weight DIA
SUBJFASTR [c]	Subject Full Analysis Set - TR Phase	Full Analysis Set Flag: This will include all patients with AD and caregivers who provide consent to release information and who fulfill the study entry criteria.<br><br>Criteria that have to be checked to include a patient in FAS:<br>Inclusion criteria:<br>1. MMSE score of 26 or less (MMSE questionnaire)<br>2. Patient is at least 55 years old (DEMO)<br>3. Written consent of both the patient (or his/her legal representative) and the caregiver; Patients are asked to give at least co-consent where able (IC)<br><br>Exclusion criteria:<br>1. Patient is simultaneously participating in another study that includes a treatment intervention and/or an investigational drug at baseline (OCT)<br>2. Stroke is reported at the baseline visit (MHC1)	AD caregivers provide consent release<br><br>Investigational drug baseline OCT stroke<br><br>Analysis set flag include patients	Subject Full Analysis Set - TR Phase, AD caregivers provide consent release. Investigational drug baseline OCT stroke. Analysis set flag include patients.
VSSTATLNM [c]	Subject Status at Visit LNM	[1] Continuing and starting clinical trial<br>[2] Continuing	Continuing discontinuing discontinuing participation clinical	Subject Status at Visit LNM, continuing discontinuing discontinuing Participation

		[3] Discontinuing<br>[4] Discontinuing due to participation in clinical trial<br><br>Missing for Visit 8	Continuing starting clinical trial continuing<br><br>Participation clinical trial missing visit	clinical. continuing starting clinical trial continuing. Participation clinical trial missing visit.
VISDTC [c]	Visit Date Character	Format as YYYY-MM-DD. If any item is missing, use dashes. Examples: 2004-01-24 2004-01---.<br><br>Missing for Visit 8	missing use dashes examples 2004<br><br>24 2004 01 missing visit<br><br>format yyyy mm dd item	Visit Date, Character missing use dashes examples 2004. 24 2004 01 missing visit. format yyyy mm dd item.
DIAGDT [c]	Disease Diagnosis Date	DIAGDT is derived using the year, month, and day date components Imputation rule:<br>If both month and day are missing, impute July 1. If only day is missing then use 15.	year month day date components<br><br>missing impute july day missing<br><br>diagdt derived using year month	Disease Diagnosis Date, year month day date components. missing impute july day missing. diagdt derived using year month.

[a] Each row in this table stands for an EU variable from the GERAS-EU cohort, with information from the first three columns provided in data dictionary.

[b] Simple concatenation case. A short derivation rule (with 20 or fewer words) is concatenated with the variable label directly.

[c] KeyBERT extraction case. When the derivation rule has more than 20 words, KeyBERT is applied to extract key words from the derivation rule and the top-3 outputs from KeyBert (each containing five key words) are concatenated with the variable label.
