# Supplementary material for: A natural language processing approach to support biomedical data harmonization: Leveraging large language models
Source: PLoS One. 2025 Jul 24;20(7):e0328262. doi: 10.1371/journal.pone.0328262 (PMC12289046; doi:10.1371/journal.pone.0328262)
Supplement: S2 Text — Additional experimental results. Table A. NLP-derived features used by the Random Forest model. Table B. Other features used by the Random Forest model. Table C. Random Forest and E5 model performance comparison. (PDF) [file pone.0328262.s002.pdf]

S2 Text. Additional experimental results

Table A. NLP-derived features used by the Random Forest model [a, b, c]

	Training set, mean (std)	Training set, range [min, max]	Test set, mean (std)	Test set, range [min, max]
MiniLM_on_label	0.16 (0.11)	[-0.21, 1]	0.15 (0.12)	[-0.16, 1]
E5_on_label	0.76 (0.03)	[0.66, 1]	0.76 (0.03)	[0.67, 1]
MPNet_on_label	0.19 (0.12)	[-0.15, 1]	0.19 (0.12)	[-0.14, 1]
BioLORD_on_label	0.12 (0.13)	[-0.25, 1]	0.12 (0.13)	[-0.28, 1]
Fuzzy_on_label	34.30 (10.54)	[0, 100]	34.68 (10.04)	[0, 100]
MiniLM_on_sheet	0.15 (0.10)	[-0.06, 0.77]	0.15 (0.10)	[-0.09, 0.77]
E5_on_sheet	0.77 (0.03)	[0.70, 0.93]	0.77 (0.03)	[0.70, 0.93]
MPNet_on_sheet	0.16 (0.10)	[-0.10, 0.74]	0.16 (0.11)	[-0.10, 0.74]
Fuzzy_on_sheet	34.49 (8.24)	[10, 100]	35.79 (11.16)	[10, 100]
BioLORD_on_sheet	0.21 (0.14)	[-0.12, 0.80]	0.21 (0.14)	[-0.12, 0.80]
MiniLM_on_label_key	0.21 (0.11)	[-0.15, 0.87]	0.20 (0.11)	[-0.19, 0.94]
E5_on_label_key	0.78 (0.03)	[0.67, 0.96]	0.78 (0.03)	[0.68, 0.97]
MPNet_on_label_key	0.26 (0.12)	[-0.14, 0.90]	0.25 (0.12)	[-0.14, 0.95]
BioLORD_on_label_key	0.14 (0.12)	[-0.23, 0.95]	0.14 (0.12)	[-0.22, 0.93]
Fuzzy_on_label_key	36.05 (9.30)	[0, 100]	36.39 (9.15)	[0, 100]

[a] NLP-derived features are similarity scores calculated by four large language models (E5, MPNet, miniLM, BioLORD) and fuzzy match using variable labels, data sheet descriptions, and key words extracted from the derivation rules. As an example, we provided the mean, standard deviation, minimum, and maximum values of these features for the training and test sets in trial 1.

[b] "on_label" in a feature name indicates similarity scores based on variable labels, "on_sheet" indicates similarity scores based on data sheet descriptions, and "on_label_key" indicates similarity scores based on the combination of variable labels and key words extracted from derivation rules.

[c] Similarity scores estimated by MiniLM, MPNet and BioLORD ranged from -1 to 1, while similarity scores generated by E5 ranged from 0.65 to 1 due to the use of a low temperature of 0.01 for infoNCE contrastive loss [1]. The fuzzy matching similarity scores ranged from 0 to 100.

Table B. Other features used by the Random Forest model

	GERAS-EU study	GERAS-JP study
**The number of words in a variable label**		
Mean (std) [a]	11.3 (8.2)	8.5 (5.6)
Median [Q1, Q3] [a]	9 [4, 18]	7 [5, 10]
**Derivation rule is empty**		
TRUE	252 (72.6%)	152 (11.5%)

FALSE	95 (27.4%)	1170 (88.5%)
**The number of key words in a non-empty derivation rule**		
Mean (std) [a]	8.8 (5.8)	11.4 (4.6)
Median [Q1, Q3] [a]	9 [3, 15]	15 [11, 20]

[a] Q1: First quantile; Q3: Third quantile; std: standard deviation.

Table C. Random Forest and E5 model performance comparison

Metric [a]	E5 [b] mean (standard deviation)	Random Forest [b] mean (standard deviation)	Mean difference [b] (Random Forest – E5), 95% confidence interval	*P* value
HR-30	0.911 (0.029)	0.987 (0.012)	0.076 [0.068, 0.085]	<0.001*
HR-20	0.905 (0.028)	0.976 (0.016)	0.071 [0.063, 0.079]	<0.001*
HR-10	0.871 (0.034)	0.928 (0.030)	0.057 [0.047, 0.066]	<0.001*
HR-5	0.803 (0.040)	0.873 (0.036)	0.070 [0.060, 0.080]	<0.001*
MRR	0.659 (0.044)	0.743 (0.037)	0.084 [0.075, 0.093]	<0.001*

[a] HR-30: Top 30 hit ratio; HR-20: Top 20 hit ratio; HR-10: Top 10 hit ratio; HR-5: Top 5 hit ratio; MRR: Mean reciprocal rank; E5: E5_large_V2 model.
[b] The performances of E5 and Random Forest compared over 50 trials, using the paired *t*-tests. The Random Forest model's hyperparameters were optimized using the MRR metric. A separate Random Forest model, optimized based on HR-30, exhibited similar performance across all metrics (see Table 4 in the main text).

References:

1. Manna S, Chattopadhyay S, Dey R, Bhattacharya S, Pal U. DySTreSS: Dynamically Scaled Temperature in Self-Supervised Contrastive Learning. 2023:arXiv:2308.01140. doi:10.48550/arXiv.2308.01140 Accessed August 01, 2023. https://ui.adsabs.harvard.edu/abs/2023arXiv230801140M
